# The differential effect of two cereal foods on gut environment: a randomized, controlled, double-blind, parallel-group study

**DOI:** 10.3389/fnut.2023.1254712

**Published:** 2024-02-22

**Authors:** Yohsuke Yamauchi, Hirofumi Masutomi, Katsuyuki Ishihara, Tenagy Hartanto, Chol Gyu Lee, Shinji Fukuda

**Affiliations:** ^1^Metagen Inc., Tsuruoka, Japan; ^2^Research and Development Division, Calbee, Inc., Utsunomiya, Japan; ^3^Institute for Advanced Biosciences, Keio University, Tsuruoka, Japan; ^4^Gut Environmental Design Group, Kanagawa Institute of Industrial Science and Technology, Kanagawa, Japan; ^5^Transborder Medical Research Center, University of Tsukuba, Tsukuba, Japan; ^6^Laboratory for Regenerative Microbiology, Juntendo University Graduate School of Medicine, Tokyo, Japan

**Keywords:** fruit granola, corn flakes, gut microbiota, defecation, dietary fiber, short-chain fatty acids

## Abstract

**Background and aims:**

Cereal-based foods such as fruit granola (FG) and corn flakes (CF) form part of a fiber-rich diet. Dietary fiber has a good effect on human health. However, changes in gut microbiota and intestinal immunity have not been investigated. We conducted a randomized, double-blind, placebo-controlled trial to investigate the effects of FG and CF intake on gut microbiota, metabolome, and the immune system.

**Methods:**

Subjects continuously consume CF or FG for 4 weeks. Stool samples, and questionnaires on defecation were collected before, 2 weeks after, and 4 weeks after intake. Gut microbiota was analyzed using 16S rRNA gene amplicon sequencing. Fecal metabolomes were analyzed using GC/MS and CE-TOF/MS. Fecal IgA was analyzed using ELISA.

**Results:**

The defecation frequency after cereal based food intake was improved. The different cereal-based foods had different effects on gut microbiome. The increase in intestinal IgA levels was positively correlated with the relative abundance of *Dialister* and the Lachnospiraceae ND3007 group in CF and FG group, respectively. SCFAs showed a positive correlation with *Prevotella* 9 in the FG group.

**Conclusion:**

This study showed that the supplement in dietary fiber contained in CF and FG improves bowel movements. CF and FG each had different effects on gut microbes, metabolites and different relationships between fecal IgA or SCFAs and gut microbiota.

## 1 Introduction

Cereals are commonly consumed worldwide, with the increasing westernization of diets in Japan, the consumption of diverse cereals, primarily during breakfast has risen (1). The widespread acceptance of cereals can be attributed to their ease of preparation and consumption. From a nutritional standpoint, regular cereal consumption can be beneficial. Individuals who consistently consume breakfast cereals exhibit higher daily intakes of dietary fiber, B vitamins, folic acid, and minerals as calcium, iron, magnesium, and zinc (2). Corn flakes and fruit granola is one of the major cereals in Japan. Constipation is a common intestinal disorder and reduces quality of life (3). Dietary fiber is one of the solutions for improving constipation (4).

Corn flakes (CF), which consists almost entirely of corn-derived dietary fiber, while FG contains a mix of dietary fibers from oats, rye, dried fruits, and nuts. We have previously reported that FG consumption for breakfast enhances bowel movements in adult women and elementary school children (1, 5). It also leads to reduced blood pressure, urinary toxin indoxyl sulfate, and improved stool characteristics for hemodialysis patients (6). Several dietary fibers can potentially influence the intestinal microbiota (5). A study looking at 24-h food records and data from fecal shotgun metagenomic analysis of 34 healthy humans collected daily over a 17-day period suggests that daily fluctuations in the microbiome are associated with food selection (7). This finding suggested that both the fiber content and diversity may have influences on the gut microbiome. They affect not only microbiome composition, but also the concentration of short-chain fatty acids (SCFAs) derived from gut microbes (8, 9). SCFAs modulate immunoglobulin A (IgA) production in the intestinal mucosa, a defense system against exogenous pathogenic bacteria. Secretory IgA (SIgA) secreted at the mucosal surface of the intestinal tract is known to form dimers, which maintain homeostasis in the intestinal tract through promotion of colonization by commensal bacteria and nonspecific labeling against exogenous pathogenic bacteria. Therefore, variety of dietary fiber intake is thought to be closely related to modulation of intestinal microbiota and the immune mechanism mediated by IgA via SCFAs. Several studies have evaluated the effects of individual cereals, but the effects of two different cereals on defecation, fecal IgA level and the intestinal environment have not been investigated.

In this study, we examined the relationship between the diversity of dietary fiber content in cereals and the concentrations of IgA derived from gut microbes. Because CF and FG contain different amounts and types of dietary fiber, their effects on IgA concentration from gut microbiota of the Japanese people are expected to be different. Moreover, the response to food intake varies from subject to subject. One factor that might account for this variation is gut microbiota composition (10, 11). It is expected that the effects of different cereal types on IgA may be different among individuals, we also examined the microbes and metabolites associated with these individual differences. For this purpose, we conducted a randomized, double-blind, placebo-controlled parallel group trial.

## 2 Materials and methods

### 2.1 Ethics approval

Human rights of all participants in this study were strictly protected at all times. The study was in accordance with the Helsinki Declaration, and Ethical Guidelines on Epidemiological Research in Japan, for clinical trials of drugs. We obtained informed consent from all participants prior to the trial, as recorded in the text. This trial was conducted following approval of the clinical trial ethics review committee of Chiyoda Paramedical Care Clinic (Approval date: 16, April 2021). The study protocol was registered with the University Hospital Health Information Network Clinical Trials Registry System (UMIN-CTR, trial number: UMIN000044381).

### 2.2 Study design

A randomized and controlled parallel trial with two 4-week dietary intervention periods was conducted. The test foods were CF and FG (Calbee, Inc., Tokyo, Japan). The subjects consumed 57.3 g of CF or 50 g of FG with 200 mL of milk per day, both with a calculated energy intake of 341 kcal. The primary outcome was the amount of intestinal IgA, with gut microbiome and metabolome composition, and defecation data we could collect (frequency, amount, consistency, color, odor, feeling of incomplete evacuation, and abstinence) secondary outcomes. At each time point, subjects were asked to collect fecal samples; (1) prior to starting cereal intake, (2) 2 weeks after starting cereal intake, and (3) 4 weeks after intake. Collected fecal samples were preserved at −20°C until processing.

**Figure 1:**
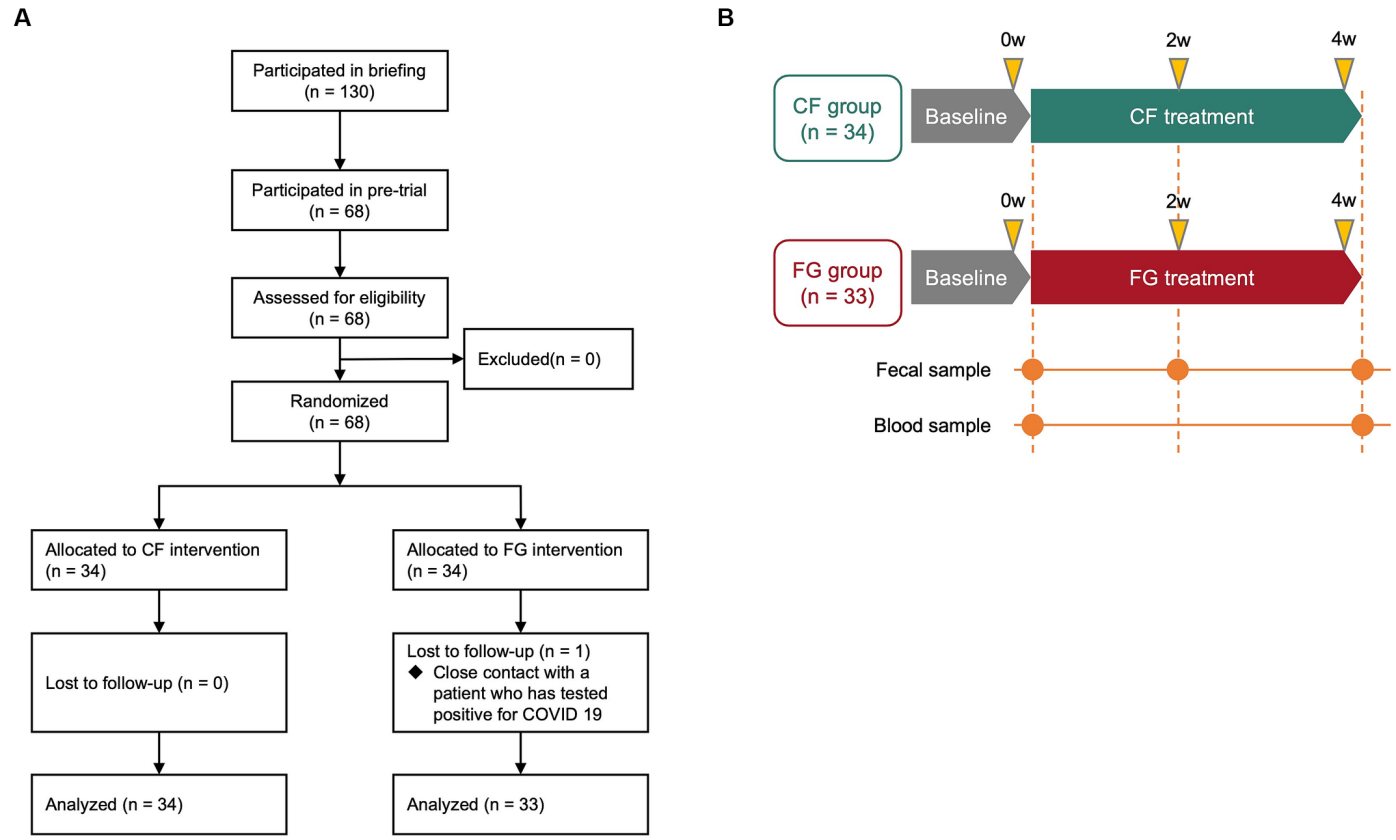
Schematic overview of the clinical trial in this study. **(A)** Flow diagram of this trial. **(B)** Clinical trial diagram and description of time points. Subjects were assigned to either the CF group or the FG group, and they consumed the assigned food for 4 weeks. CF; corn flake, FG; fruit granola.

Out of 130 subjects recruited for pre-trial, 68 subjects among them who met the following criteria were enrolled for this trial (Figure 1A): (1) males and females aged 20–59 years old; (2) those able to have breakfast regularly, and replace their usual breakfast with the test food; (3) those with defecation frequency of 3–7 times per week; (4) those who show understanding of the study procedures and agree to participate in the study, by written informed consent prior to the study. Key exclusion criteria are described in Supplementary Data 1.

These 68 subjects were divided into two groups (the CF group and the FG group) through stratified random sampling taking into consideration age, the male–female ratio, defecation frequency, and stool amount. Next, a test food assignment table with a subject identification code was prepared. Immediately after the assignment to the test food group according to the assignment table, the table was sealed and kept tightly concealed by the subject assignment manager. The table was disclosed to the test analyst, investigator, and test sharing doctor after data fixation.

### 2.3 Measurement of clinical data

Body composition and blood samples were collected at baseline and after 4 weeks of the dietary intervention period. The health data such as body water, muscle mass, and fat mass, was measured using InBody 410 (InBody Japan Inc., Japan) (12, 13).

### 2.4 Questionnaire survey on defecation status

The following 8 items regarding bowel movements were surveyed at 3 time points: 0 weeks, 2 weeks, and 4 weeks after the start of the trial. Stool consistency was rated on a seven-point scale: 1, very hard; 2, hard; 3, somewhat hard; 4, normal; 5, somewhat soft; 6, soft (muddy); 7, very soft (watery). Stool color was rated on a six-point scale: 1, yellow; 2, dark yellow; 3, ochre; 4, brown; 5, dark brown; 6, blackish brown. Fecal odor was rated on a five-point scale: 1, very weak; 2, weak; 3, distinct; 4, strong; 5, very strong. Feeling of incomplete defecation was rated on a four-point scale: 1, no residual stool feeling and very refreshing; 2, almost no residual stool feeling and somewhat refreshing; 3, slightly feeling residual bowel movement; 4, unpleasant with a lingering stool sensation. Abdominal pain was rated on a four-point scale: 1, a strong pain; 2, a weak pain; 3, almost no pain; 4, no pain at all. Defecation frequency was analyzed based on two items: bowel movement counts/days and days with bowel movement/total days.

### 2.5 Processing and analysis of fecal samples

DNA extraction from fecal samples was performed as previously reported (14). The fecal samples were initially lyophilized and shaken vigorously using a Shake Master (1,500 rpm, 10 min; Biomedical Science Co.) Samples were then suspended in DNA extraction buffer containing 400 μL of a 1% w/v SDS/TE (10 mM Tris–HCl, 1 mM EDTA; pH 8.0) solution, and fecal samples in the buffer were further shaken with 0.1 mm zirconia/silica beads using a Shake Master (1,500 rpm, 5 min). After centrifugation (17,800 × g; 10 min; room temperature), bacterial DNA was extracted using an automated DNA extraction machine (GENE PREP STAR PI-480). Amplification of 16S rRNA gene was carried out with forward and reverse primers, respectively, (27F-mod; AGRGTTTGATYMTGGCTCAG, 338R; TGCTGCCTCCCGTAGGGAGT) (14). The amplified DNA was sequenced using MiSeq (Illumina, San Diego, CA, United States) according to the manufacturer’s protocols.

### 2.6 Metabolome analysis

Metabolites were extracted from fecal samples as previously described (15). Metabolites except for acetic acid were measured with capillary electrophoresis time-of-flight mass spectrometry (CE-TOF/MS). Acetic acid, which cannot be measured by CE-TOF/MS because it is contained in running solution, was measured with gas chromatography mass spectrometry (GC/MS; 7890B, 5977B; Agilent Technologies, Inc.) as previously described (16) with some modifications. Acetic acid were extracted from a total of 0.05 g of human feces by shaking with 0.4 mL of diethyl ether and 0.2 mL of chloroform (FUJIFILM Wako Pure Chemical Corp., Osaka, Japan) and then acidified with 0.05 mL of 1 mol/L. Then, 0.3 mL of supernatant was mixed with 0.1 mL of derivatization and 1 mL of derivatization reagent (TMSI-H: 76% pyridine, 16% hexamethyldisilazane, and 8% trimethylchlorosilane), GL Science Inc. After heating, the sample was placed on ice for 10 min and centrifuged at 14,000 rpm at room temperature for 30 s. A total of 2 μL of the organic phase was injected into the capillary column (InertCap Pure WAX (30 m × 0.25 mm, df = 0.5 um); GL Science Inc.) The initial temperature was 80°C and the final temperature was 200°C. Helium was used as carrier gas.

Metabolome analysis with CE-TOF/MS was conducted according to HMT’s *Basic Scan* package, using CE-TOF/MS based on the methods described previously (17, 18). Lyophilized feces were extracted with vigorous shaking in 500 μL of methanol and internal standards (20 μM of methionine sulfone and 20 μM of D-camphor-10-sulfonic acid). The samples were disrupted by intense shaking with 0.1 mm zirconia/silica beads (1,500 rpm, 5 min). Following the addition of 200 μL of ultrapure water and 500 μL of chloroform, samples were further centrifuged at 4,600 × g for 15 min and 150 μL of the aqueous layer was transferred to a centrifugal filter tube (Ultrafree MC-PLHCC 250/pk for Metabolome Analysis, Human Metabolome Technologies, Yamagata, Japan). The filtrate was centrifuged and dissolved in 50 μL of ultrapure water immediately before capillary electrophoresis coupled with electrospray ionization time-of-flight mass spectrometry (CE-TOF/MS; Agilent Technologies, Inc., Santa Clara, CA, United States) analysis. Briefly, CE-TOF/MS analysis was carried out using an Agilent CE capillary electrophoresis system equipped with an Agilent 6210 time-of-flight mass spectrometer (Agilent Technologies, Inc., Santa Clara, CA, United States). The systems were controlled by Agilent G2201AA ChemStation software version B.03.01 (Agilent Technologies) and connected by a fused silica capillary (50 μm *i.d.* × 80 cm total length) with commercial electrophoresis buffer (H3301-1001 and I3302-1023 for cation and anion analyses, respectively, HMT) as the electrolyte. The spectrometer was scanned from *m/z* 50 to 1,000 and peaks were extracted using MasterHands, automatic integration software (Keio University, Tsuruoka, Yamagata, Japan) in order to obtain peak information including *m/z*, peak area, and migration time (MT) (19). Signal peaks corresponding to isotopomers, adduct ions, and other product ions of known metabolites were excluded, and the remaining peaks were annotated according to HMT’s metabolite database based on their *m*/*z* values and MTs. Areas of the annotated peaks were then normalized to internal standards and sample amount in order to obtain relative levels of each metabolite. Relative peak area was calculated following identification of the peak from CE-TOF/MS data. Calculation of the relative peak area was done by dividing the peak areas of individual metabolites by that of the internal standards. Of the 432 metabolites for which relative peak area was calculated, the quantitative value of 119 metabolites were determined through comparison with the reference material.

### 2.7 IgA amount in fecal samples

IgA Extraction of fecal IgA was performed as previously reported (20). Quantification of IgA amount in fecal samples was performed by ELISA using commercially available kits according to the manufacturer’s protocols (Human IgA ELISA Kit, Abcam ab196263).

### 2.8 Bioinformatics analysis

QIIME2 version 2019.10 was used for the 16S rRNA gene-based microbiome analysis (21). The primer bases were deleted using cutadapt (options: -p-discard-untrimmed) (22). Subsequently, DADA2 was used for de-noising, quality filtering and generating amplicon sequence variants (ASVs) (options: -p-trunc-len-f 230 -p-trunc-len-r 130) (23). The ASVs were assigned to taxa by applying a classifier for Silva SSU Ref Nr 99 (version 132) (command: “qiime feature-classifier classify-sklearn”; options: default) (24).

### 2.9 Statistical analysis

The statistical analyses were performed using Python scripts (version 3.7.6). For beta-diversity analysis, microbiome weighted UniFrac distance and metabolome Bray-Curtis distance were used. Distance matrices were visualized via principal coordinate analysis (PCoA) analysis, and groups and time points were compared with PERMANOVA. Wilcoxon signed-rank test was used (scipy version 1.5.2) for the comparison of each microbe and metabolite. The false discovery rate (FDR) was corrected with the Benjamini–Hochberg method (statsmodels version 0.10.0). Microbes with a mean relative abundance below 0.001 and metabolites undetected in 75% of the samples were excluded for comparison.

### 2.10 Individual effects for microbiome, metabolome, and IgA to cereal consumption

We used spearman’s and pearson’s correlation analysis methods to evaluate individual effects of cereal consumption. We defined the test food effect size as the differential value and used it to evaluate whether effects depended on individual basal characteristics. The differential value was calculated with the following equation:


$$\mathrm{Differential}\;\mathrm{value}=\left(V2-V1\right)/V1$$

V1:valuebeforeintake

V2:valueafterintake

We presented two types of feature values: baseline value and differential value. If a significant correlation is observed between baseline value and IgA, it may indicate intestinal factors that are responsive to cereal intake. If significant correlation is found between differential value and IgA, it indicates the groups of bacteria or metabolites that contributed to the increase in IgA.

## 3 Results

### 3.1 Overview of the clinical trial

We conducted a randomized, double-blind, controlled parallel-group study to quantify the effects of intake of CF and FG on the gut environment (Figures 1A,B). In total, 67 subjects completed the trial; one subject in the FG group was dropped as tested positive for COVID-19. All 67 subjects who completed the study had no adverse events (Figure 1A). At baseline, the CF group consisted of 18 males and 16 females, with a mean age of 43.6 ± 9.1 years, mean Body Mass Index (BMI) of 21.1 ± 2.2, mean frequency of bowel movements of 0.8 ± 0.2 times/day, and mean stool volume of 2.3 ± 1.1 pieces per day (Table 1). The FG group consisted of 17 males and 16 females, with a mean age of 43.9 ± 11.3 years, mean BMI of 21.6 ± 2.6, mean frequency of bowel movements of 0.8 ± 0.2 times/day, and mean stool volume of 2.3 ± 1.1 pieces/day (Table 1).

**Table 1:** Subject information.

	CF	FG	*p*-value
Subjects	34	33	
Sex (male, female)	18, 16	17, 16	–
Age (years)	43.6 ± 9.1	43.9 ± 11.3	0.93
BMI (kg/m)^2^	21.1 ± 2.2	21.6 ± 2.6	0.45
Defecation frequency (times/day)	0.8 ± 0.2	0.8 ± 0.2	0.71
Stool amount	2.3 ± 1.1	2.3 ± 1.1	0.93

Values are means ± standard deviation (SD). Stool amounts were determined by counting the number of eggs.

### 3.2 The effect of cereal-based foods on defecation and fecal IgA

**Figure 2:**
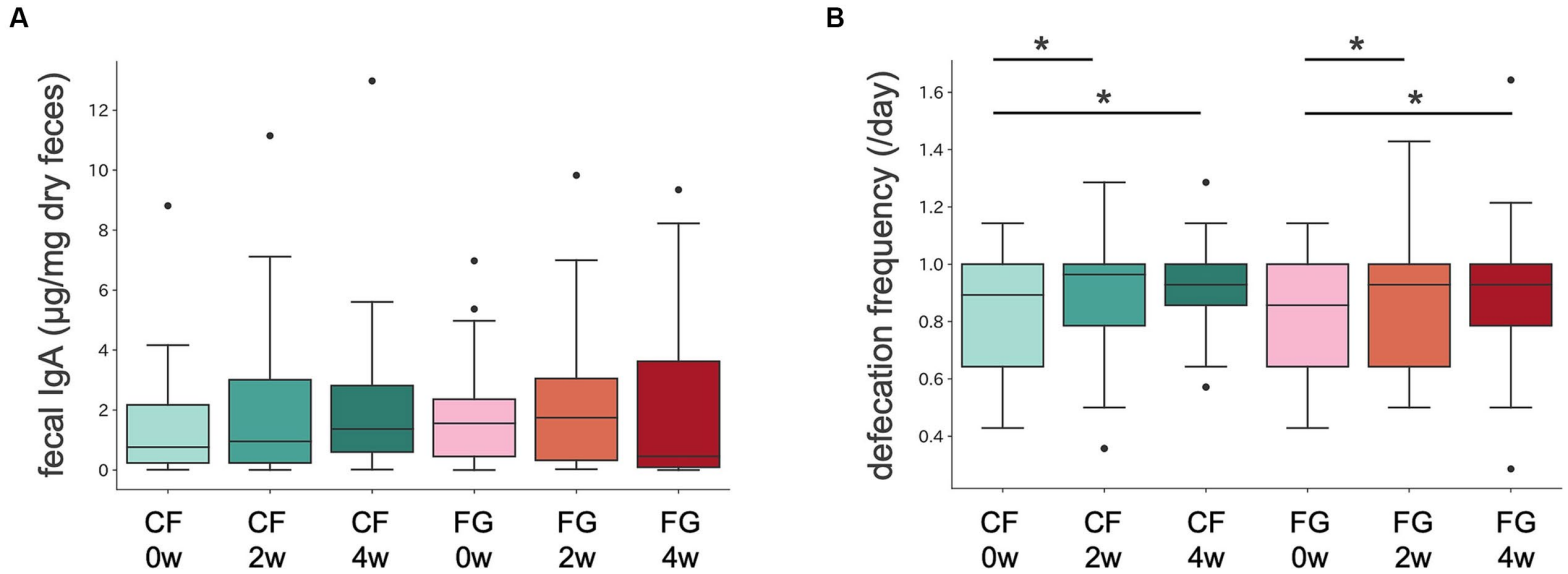
Intestinal IgA content and defecation frequency in CF and FG groups. Distribution of **(A)** intestinal IgA content and **(B)** defecation frequency. The Wilcoxon signed-rank test was performed for comparisons between groups at The Wilcoxon signed-rank test was performed for comparisons between groups at each intake period (0, 2, or 4 weeks). The Wilcoxon signed-rank test was performed for comparisons between groups at each intake period (2 or 4 weeks). CF; corn flake, FG; fruit granola.

Stool properties as stool amount, consistency, and color were not significantly different between in the CF and FG group. Defecation frequency increased in both the CF and FG groups 2 and 4 weeks after intake compared to before intake (Figure 2B; Table 2).

**Table 2:** Stool condition, IgA amount and defecation frequency in CF and FG groups.

	CF			FG		
	0 week	2 weeks	4 weeks	0 week	2 weeks	4 weeks
Fecal IgA (mg/g dry feces)	1.37 ± 1.77	2.20 ± 2.67	2.04 ± 2.43	1.90 ± 1.79	2.14 ± 2.26	2.02 ± 2.68
Stool amount	2.31 ± 1.15	2.34 ± 1.21	2.35 ± 1.46	2.29 ± 1.12	2.32 ± 1.24	2.36 ± 1.18
Stool consistency	3.79 ± 0.80	3.74 ± 0.57	3.89 ± 0.54	3.71 ± 0.65	3.78 ± 0.60	3.83 ± 0.64
Stool color	3.93 ± 0.80	3.93 ± 0.83	3.83 ± 0.75	3.94 ± 0.77	3.89 ± 0.75	3.93 ± 0.73
Fecal odor	3.02 ± 0.26	3.06 ± 0.25*	3.02 ± 0.28	3.00 ± 0.40	2.96 ± 0.41	2.95 ± 0.43
Feeling of incomplete evacuation	1.90 ± 0.50	1.91 ± 0.55	1.86 ± 0.56	2.20 ± 0.63	2.14 ± 0.59	2.11 ± 0.60
Abdominal pain	3.47 ± 0.61	3.47 ± 0.55	3.48 ± 0.52	3.33 ± 0.66	3.36 ± 0.63	3.38 ± 0.58
Defecation frequency (bowel movement counts/days)	0.83 ± 0.19	0.88 ± 0.21*	0.91 ± 0.17*	0.81 ± 0.21	0.87 ± 0.23*	0.90 ± 0.26*
Defecation frequency (days having a bowel movement/total days)	11.29 ± 2.75	11.53 ± 2.73	11.91 ± 2.35	10.67 ± 2.85	11.09 ± 2.49	11.52 ± 2.72*

Values are means ± SD. Stool amounts were determined by counting the number of eggs. *p < 0.05, Wilcoxon signed-rank test. CF; corn flake, FG; fruit granola.

Stool IgA levels did not significantly change at 2 or 4 weeks after consumption compared to before consumption in both the CF and FG groups (Figure 2A; Table 2).

### 3.3 Effect of cereal-based foods intake on the gut microbiome and metabolome

**Figure 3:**
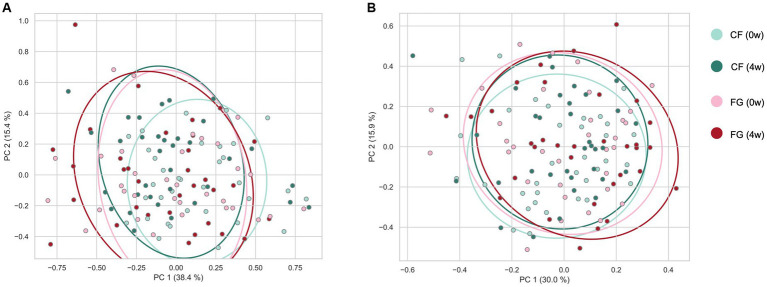
Effect of cereal-based food consumption on gut microbiome and metabolome profiles. The **(A)** weighted UniFrac distance for the gut microbiome and **(B)** Bray–Curtis distance for quantitative intestinal metabolome profiles among all samples. CF; corn flake, FG; fruit granola.

We investigated the changes of intestinal microbiota and metabolites by cereal intake. In total, 231 bacteria and 432 metabolites were detected. Of these, quantitative values of 120 metabolites in the fecal samples (Supplementary Table S2) were also determined. According to beta diversity analysis, the weighted UniFrac distance of the gut microbiota did not change significantly before and after intake in both the CF and the FG groups (Figure 3; Table 3; *p* < 0.05; PERMANOVA). In contrast, the Bray–Curtis distance of the metabolome showed that significant changes were observed before and after FG intake.

**Table 3:** Result of permutational analysis of variance (PERMANOVA).

	CF	FG
**Microbiome weighted UniFrac**		
*p*-value	0.058	0.956
R2 score	0.060	0.010
**Metabolome Bray–Curtis**		
*p*-value	0.715	0.017
R2 score	0.020	0.074

PERMANOVA was calculated in each group between 0w and 4w. CF; corn flakes, FG; fruit granola.

**Figure 4:**
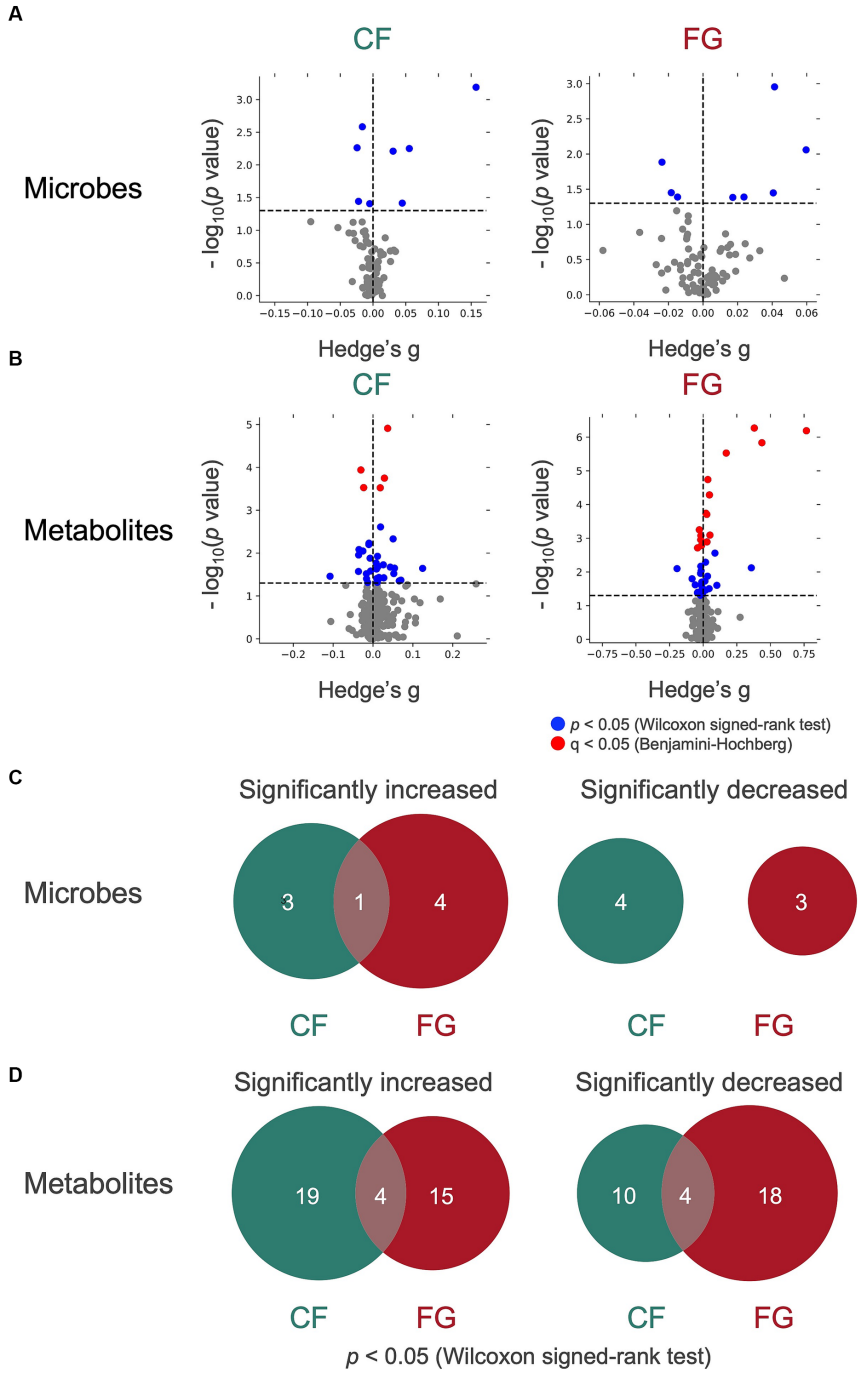
Effect of cereal-based foods intake on gut microbes and metabolites. **(A,B)** The volcano plot of the effect on **(A)** microbes and **(B)** metabolites of cereal intake. The *y-axis* represents the logarithm of the Wilcoxon signed-rank test *p*-value compared with the baseline. lt; 0.05 in *y-axis*, the point is shown in blue; if *q* < 0.05 in *y-axis*, the point is shown in red; otherwise, the point is shown in gray. **(C,D)** The Venn diagram of **(C)** microbes and **(D)** metabolites that show significant increase or decrease. CF; corn flake, FG; fruit granola.

We proceeded to analyze the relative abundance of each bacterium amount of each metabolite in feces (represented by the scaled peak areas of each metabolite) to assess the effect of cereal intake on each bacterium and metabolite (Figures 4A,B). The relative abundance of four genera (*Bifidobacterium, Subdoligranulum, Ruminococcus* 2*, Collinsella*) significantly increased in the CF group, whereas five bacterial genera (*Parabacteroides, Mitsuokella, Ruminiclostridium* 5*, Veillonella, Collinsella*) significantly increased in the FG group (Figure 4C; Table 4; *p* < 0.05; Wilcoxon signed-rank test). The common change for these two groups is the significant increase in *Collinsella*. On the other hand, a significant decrease in the relative abundance of four bacterial genera (*Lachnospira*, *Lachnoclostridium*, *Phascolarctobacterium*, Lachnospiraceae ND3007 group) in the CF group and three bacterial genera (*Dorea, Faecalitalea, Odoribacter*) in the FG group was observed (Figure 4C; Table 4; *p* < 0.05; Wilcoxon signed-rank test).

**Table 4:** Significantly different gut microbes in CF and FG groups.

CF				FG			
*Microbes*	Effect size	*p*-value	*q*-value	*Microbes*	Effect size	*p*-value	*q*-value
**Increased microbes**							
*Bifidobacterium*	0.157	0.001	0.053	*Ruminiclostridium 5*	0.041	0.001	0.091
*Collinsella*	0.031	0.006	0.101	*Parabacteroides*	0.060	0.009	0.357
*Ruminococcus 2*	0.055	0.006	0.101	*Mitsuokella*	0.041	0.036	0.424
*Subdoligranulum*	0.045	0.038	0.402	*Collinsella*	0.024	0.041	0.424
				*Veillonella*	0.017	0.041	0.424
**Decreased microbes**							
*Phascolarctobacterium*	−0.016	0.003	0.101	*Dorea*	−0.024	0.013	0.357
*Lachnoclostridium*	−0.024	0.005	0.101	*Faecalitalea*	−0.018	0.035	0.424
*Lachnospira*	−0.022	0.036	0.402	*Odoribacter*	−0.015	0.041	0.424
*Lachnospiraceae ND3007 group*	−0.005	0.039	0.402				

In total, 23 metabolites were significantly increased in the CF group and 18 in the FG group, while the relative area of 14 metabolites in the CF group and 23 in the FG group decreased significantly (Figure 4D; Supplementary Table S3). Four of these metabolites were common to both groups. There was no significant change of SCFAs (acetic acid, propionic acid, butyric acid). However, some subjects showed an increase in SCFAs. Acetic acid increased in 20 subjects in the CF group and 14 subjects in the FG group. Propionic acid increased in 19 subjects in the CF group while 18 subjects in the FG group. Butyric acid increased in 21 subjects in the CF group while 15 subjects in the FG group.

### 3.4 Individual difference of cereal consumption on intestinal IgA, microbes, and metabolites

**Figure 5:**
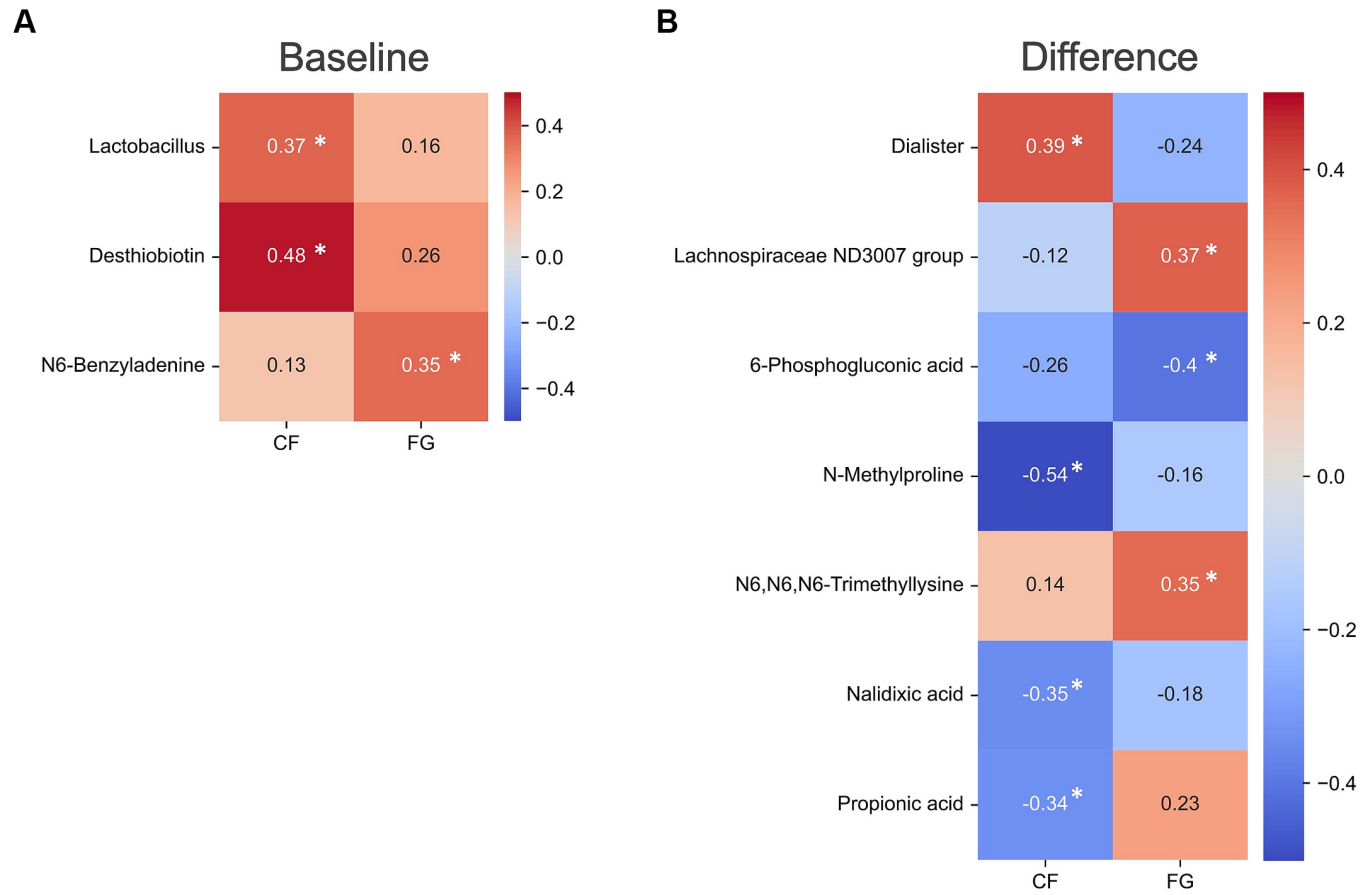
Correlation analysis of gut environment with intestinal IgA. Heatmap of the significantly correlated differential values of IgA content (4w - 0w). Colors show the Spearman coefficients, and stars show Spearman coefficients’ no-correlation test (*, *p* < 0.05). **(A)** Correlation differential value before and after intake in IgA content and baseline values of each microbe and metabolite abundance; **(B)** correlation CF; corn flake, FG; fruit granola.

Differential levels of the fecal IgA showed a positive correlation with the baseline abundance of *Lactobacillus* and one metabolite (Dasthiobiotin) in the CF group (Figure 5A; Supplementary Table S4). This suggests that individuals with elevated levels of Lactobacillus or Dasthiobiotin in their gut may exhibit an augmented IgA response upon CF intake. On the other hand, within the FG group, no significant correlation was found between IgA and the baseline levels of microbes or metabolites.

Correlation analyses between the differential levels of intestinal IgA levels and the difference levels various microbes or metabolites revealed a significant correlation between one bacterium (*Dialister*) and three metabolites (N-Methylproline, Nalidixic acid, and Propionic acid) in the CF group. Within the FG group, the bacterium Lachnospiraceae ND3007 group and two metabolites (6-phosphogluconic acid and N6, N6, N6-trimethylsine) correlated with the intestinal IgA levels (Figure 5B). These results imply the elevation of IgA following FG and CF intake might be mediated by distinct mechanisms across individual subjects.

### 3.5 Individual difference of cereal consumption on SCFAs and microbes

**Figure 6:**
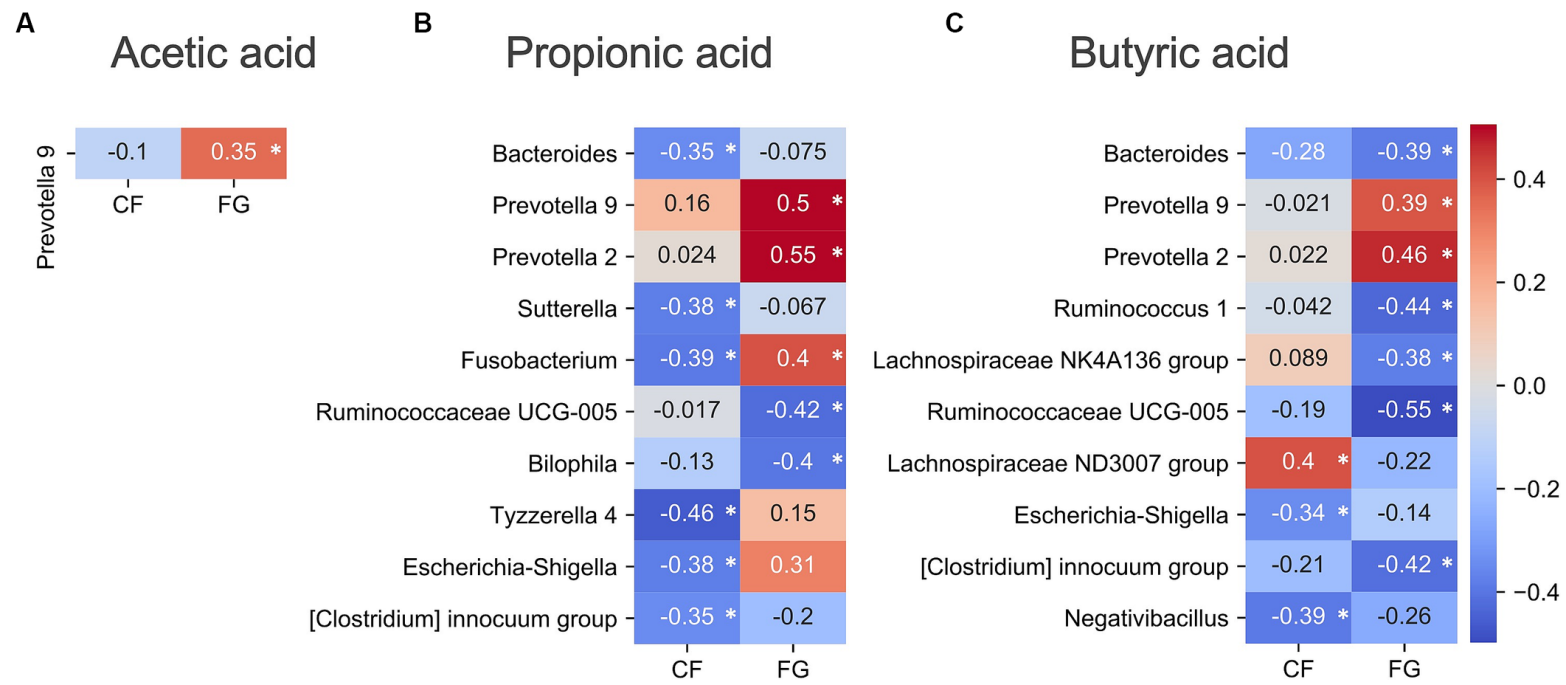
Correlation analysis of gut environment with SCFAs. Heatmap of the significantly correlated differential values of SCFAs content (4w - 0w). Acetic acid was measured by GC–MS, propionic acid and butyric acid were measured by CE-TOF/MS. Colors show the Spearman coefficients, and stars show Spearman coefficients’ no-correlation test (*, *p* < 0.05). **(A)** Acetic acid; **(B)** Propionic acid; **(C)** Butyric acid. CF; corn flake, FG; fruit granola.

In the FG group, *Prevotella* group showed significant correlation between acetic acid, propionic acid, and buryric acid (Figure 6). On the other hand, in the CF group, only Lachnospiraceae had positive correlation with butyric acid. Therefore, the response mechanisms for SCFA production of CF and GF intake are different.

## 4 Discussion

In this study, we evaluated a variety of dietary fiber on gut microbiome, metabolome, and fecal IgA. Dietary fiber is known to improve constipation (4).

Defecation frequency significantly increased in both the CF and the FG groups after intake of the test food compared to before intake (Figure 2B). Dietary fiber has viscosity and water retention-enhancing properties, which may work to increase stool bulk and lubricate transport in the intestine (25). The promotion of defecation by fiber-rich foods has been confirmed by previous studies in adults (26). CF and FG contained 2.7 g and 4.5 g of dietary fiber each, suggesting that dietary fiber intake may have improved defecation frequency. The microbiome analysis revealed that *Bifidobacterium* increased in the group of CF. The increase in frequency of bowel movements via consumption of CF may not only be due to dietary fiber supplementation, but also to the possibility that *Bifidobacterium* improves bowel movements through acetic acid production.

It has been reported that the same amount of FG as this trial intake improves the constipation of healthy Japanese women and dialysis patients (3). This suggests that the increase in defecation frequency with FG intake is a reproducible result. In addition, the FG group showed a decrease in abdominal distention (Table 3). Whole grain rye has been reported to reduce bloating compared to refined wheat (27). Rye flower was also contained in the FG used in this study, and it might contribute to reduced bloating. Cereal-based foods such as FG and CF can be solutions for dietary fiber deficiency and constipation.

Focusing on the gut bacteria that were uniquely altered in the CF group, *Bifidobacterium*, *Subdoligranulum*, and *Ruminococcus* 2 significantly increased, while *Lachnospira*, *Lachnoclostridium*, *Phascolarctobacterium*, and Lachnospiraceae ND3007 were significantly decreased. In a previous study, arabinoxylan from corn was reported to increase *Bifidobacterium* (28, 29). Corn bran consists of 51% Arabinoxylan (3). CF used in this study also contains arabinoxylan, which could increase the abundance of *Bifidobacterium.* Furthermore, *Bifidobacterium* produces acetic acid and contributes to the prevention of *E. coli* O-157:H7 infection and improvement of bowel movements (30, 31). *Subdoligranulum*, which was revealed to increase with CF intake, is involved in the production of butyric acid (32). *Subdoligranulum* sp. has been implicated in the degradation of arabinoxylan with *Bifidobacterium longum* (28). In contrast, eight bacterial genera were significantly altered in the FG group, including *Parabacteroides and Ruminiclostridium* 5, which are known fiber-degrading bacteria.

The metabolome profile changed in the FG group between before and after intervention, while it did not change in the CF group. In the FG group, the amount of saturated fatty acids in stools increased after 4 weeks of intake. Since most of them were saturated fatty acids contained in FG, it is possible that these saturated fatty acids were not the result of metabolism by gut microbiota, but the substances contained in the test food. Focusing on the individual metabolites, some showed significant variations common to CF and FG, but many showed significant variations specific to each food. Of the four substances commonly increased (terephthalic acid, 2-Carboxybenzaldehyde, S-Adenosylmethionine, and 5-Oxohexanoic acid), no previous study directly mentioned an increase due to corn consumption. However, S-Adenosylmethionine biosynthesis has been suggested to be enhanced by consuming dietary fats and oils, including corn oil (33).

Increasing dietary fiber intake has been shown to increase the production of SCFAs and IgA in the gut (34, 35). SCFAs are produced by the fermentation of dietary fiber by gut microbiota and have been associated with various health benefits.

The observed changes in gut SCFAs and fecal IgA levels between pre- and post-intervention were not statistically significant for both the CF and FG cohorts. Nevertheless, a spectrum of individual responses was discerned, with some participants showing an elevation and others a decline in IgA levels post-consumption across both dietary interventions. The inherent variability in individual health responses to functional foods remains a significant concern, as documented in references (36, 37). These variations can influence individual responses, categorizing some as “responders” who exhibit the anticipated effects, while others as “non-responders.” For instance, in a study involving mice fed barley, an elevation in *Prevotella* was detected among responders, where improved glucose tolerance was noted. This change was attributed to the production of succinic acid by *Prevotella*, resulting from the decomposition of β-glucan present in barley, which subsequently modulated glucose metabolism in the liver (38). To further elucidate the characteristics of the intestinal environment in subjects with heightened IgA and to discern potential similarities or differences between the CF and FG cohorts, we undertook a correlation analysis linking fecal IgA levels with the intestinal milieu.

In the correlation analysis of increased fecal IgA levels, changes in Lachnospiraceae ND3007 group in the FG group were positively correlated with changes in the fecal IgA level. The test food, FG, actually contains rye (Table 5). Lachnospiraceae ND3007 group is reported to be increased by rye intake and produce butyric acid (39). Butyric acid is known to promote IgA production in the intestine. Since FG contains rye flour, it is possible that FG intake increases the abundance of Lachnospiraceae ND3007 group and promotes the production of IgA in the gut through butyric acid production. However, no relationship was found between changes in butyrate and fecal IgA levels in this study (Supplementary Figure S1), mainly because butyrate produced in the intestinal tract was absorbed by intestinal epithelial cells.

**Table 5:** Composition of test foods.

	CF	FG
Energy (kcal)	341	341
Protein (g)	8.7	10.7
Fat (g)	8.5	15.1
Sugar (g)	58.9	41.1
Dietary fibers (g)	2.7	4.5
Salt (g)	0.9	0.4
Ingredients	Corn grits, sugar, syrup, salt, malt extract, emulsifier	Oats, rye flour, sugar, dried fruits (papaya, raisins, apples, strawberries), wheat flour, coconut, maltodextrin, vegetable oil, rice flour, soluble fiber, corn flour, pumpkin seeds, almond powder, salt, wheat bran, brown rice flour, apple juice, lactose, glycerin, iron citrate Na, acidifier, antioxidant (vitamin E, rosemary extract), vitamin B, vitamin C, vitamin E6, vitamin C6, vitamin C7, vitamin C8, vitamin C9, vitamin C10, vitamin C12, vitamin B12 antioxidant (vitamin E, rosemary extract), processed starch, niacin, pantothenic acid Ca, vitamin A Vitamin B6, Vitamin B1, Folic acid, Vitamin D, Vitamin B12 Vitamin B12

The production of IgA in the intestinal tract is promoted by SCFAs. In this study, SCFAs (acetic acid, propionic acid, and butyric acid) did not significantly increase in the CF and FG groups. However, individual variations revealed the presence of responders (Supplemental Table S5). Therefore, we explored the characteristics of the intestinal milieu of the responders that increase SCFAs by cereal consumption as well as IgA and compared the characteristics of the responders that increase short-chain fatty acids by CF and FG consumption.

*Prevotella* 9 showed positive correlations with the three SCFAs in the FG group; *Prevotella copri* is known to produce succinic acid (11), and in this study the rate of change in succinic acid and Prevotella 9 was positively correlated with the rate of change in succinate (*R* = 0.466, *p* = 0.006, Spearman’s coefficients; Supplementary Table S5). Succinic acid is known to be metabolized by intestinal bacteria to SCFAs, and it is possible that SCFAs increased in the FG group via an increase in Prevotella 9 and succinic acid.

## 5 Conclusion

This study indicated that the consumption of cereals such as FG and CF may provide dietary fiber and improve bowel movements in response to insufficient dietary fiber intake. FG and CF each had different effects on gut microbiota and metabolome profiles, suggesting a relationship between specific gut microbes and enhancement of IgA and SCFAs production. The effects of cereal intake on gut SCFAs and fecal IgA were different among individuals. The results suggest that this may contribute to the future development of personalized foods, in which food and fiber are selected to suit the gut microbes unique to each individual.

## Data availability statement

The data presented in this study are deposited in the DDBJ Sequence Read Archive repository, accession number: DRA016425. Clinical, gut metabolome, and gut microbiota raw data can be found in the Supplementary material. The source code used in this study is available to download here: https://github.com/metagen/article_pipeline_MGP020.

## Ethics statement

The studies involving humans were approved by Chiyoda Paramedical Care Clinic. The studies were conducted in accordance with the local legislation and institutional requirements. The participants provided their written informed consent to participate in this study.

## Author contributions

YY: Writing – original draft. HM: Writing – original draft. KI: Writing – review & editing. TH: Writing – original draft. SF: Writing – review & editing. CL: Writing – review & editing.
