# Supplementary material for: The differential effect of two cereal foods on gut environment: a randomized, controlled, double-blind, parallel-group study
Source: Front Nutr. 2024 Feb 22;10:1254712. doi: 10.3389/fnut.2023.1254712 (PMC10917986; doi:10.3389/fnut.2023.1254712)
Supplement: Supplementary file 2 [file Data_Sheet_2.docx]

**Supplementary Figure 1**

**(A)**

**
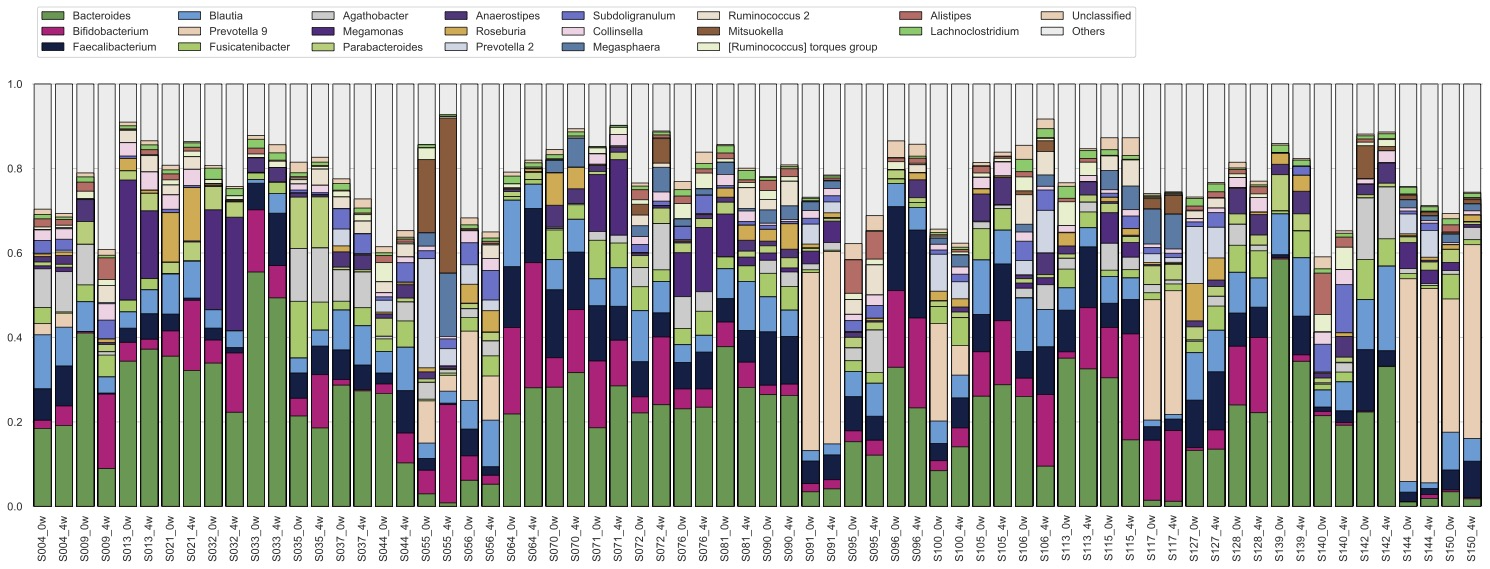
**

**(B)**

**
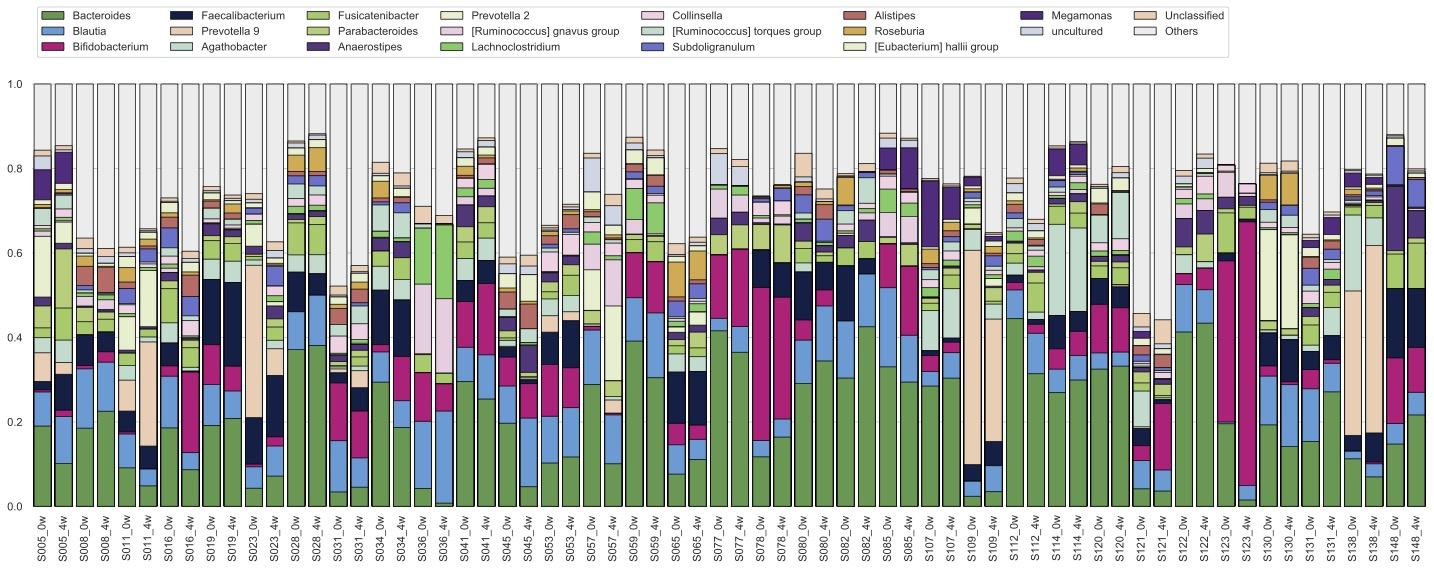
**

**(C)**

**
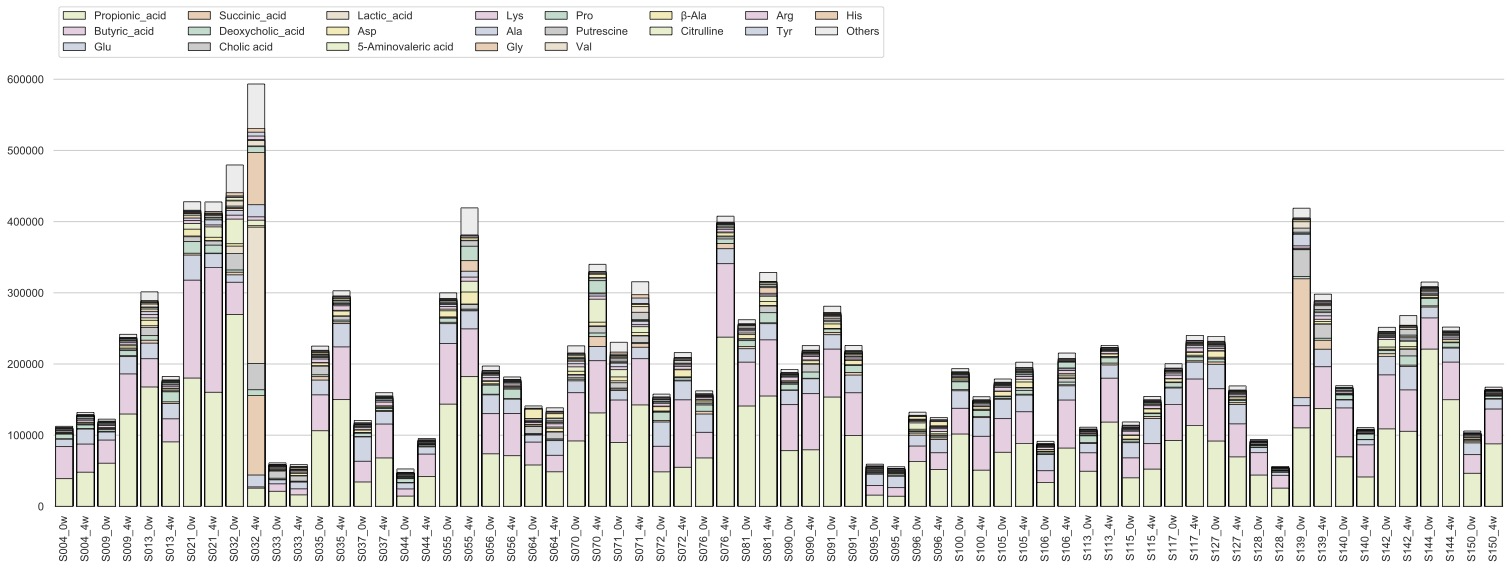
**

**(D)**

**
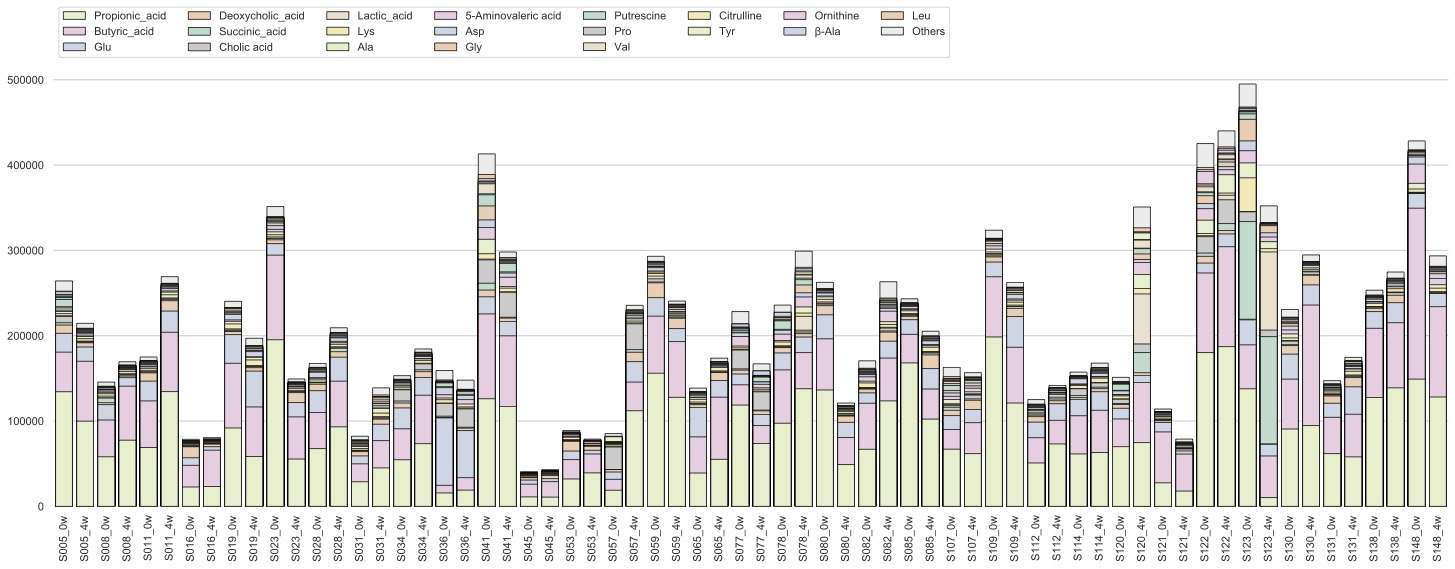
**

**The relative abundance of gut microbiomes and metabolites before and after cereal intake**

1. Changing the gut microbiome before to after CF intake
2. Changing the gut microbiome before to after FG intake
3. Changing the gut metabolome before to after CF intake
4. Changing the gut metabolome before to after FG intake

**Supplementary Figure 2**


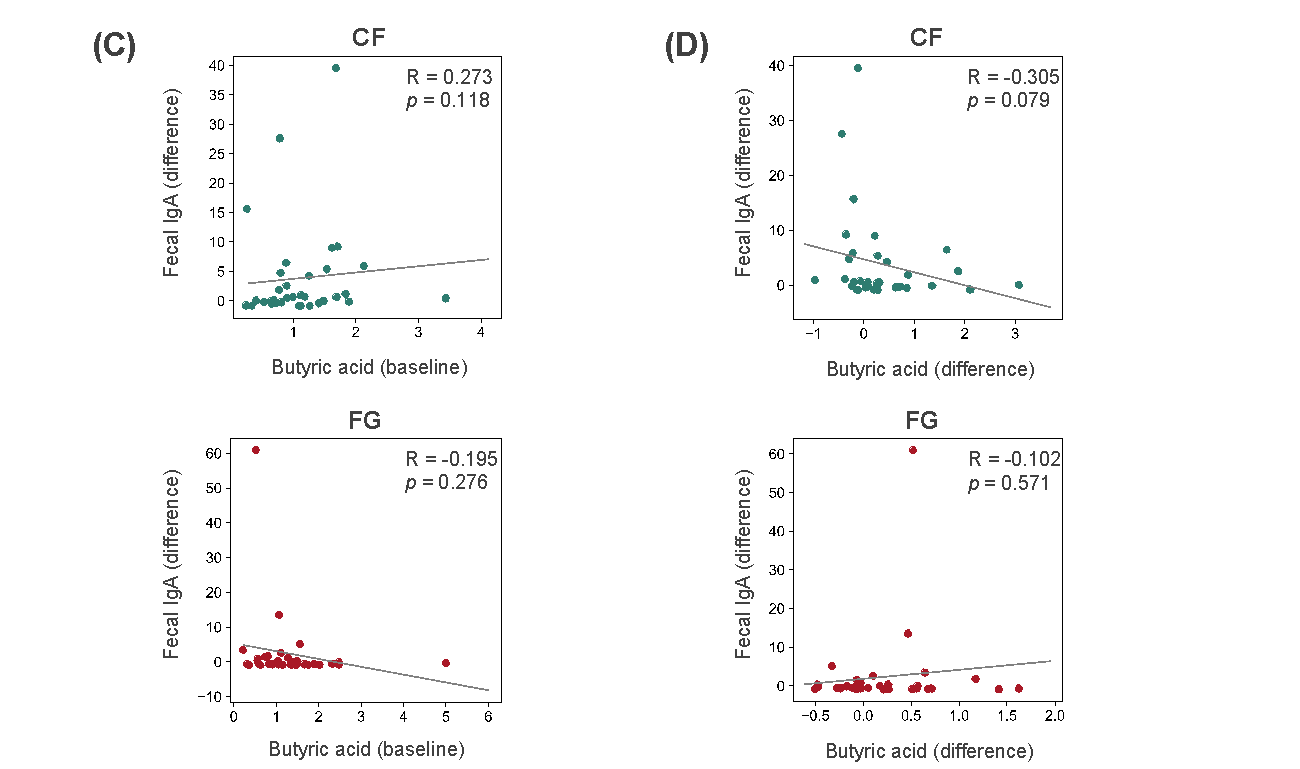


**Supplementary Figure 1. Correlation analysis with intestinal IgA and butyric acid.**

(C - D) Scatter plot of fecal IgA and Butyric acid. The y-axis represents differential values of IgA content (4w - 0w). The y-axis represents (C) baseline values and (D) differential values of Butyric acid (4w - 0w), respectively. R; Spearman coefficients, p; p-value of Spearman coefficients’ no-correlation test. CF; corn flake, FG; fruit granola.
